# Dupuytren’s Disease Predicts Increased All-Cause and Cancer-Specific Mortality: Analysis of a Large Cohort from the U.K. Clinical Practice Research Datalink

**DOI:** 10.1097/PRS.0000000000006551

**Published:** 2019-12-17

**Authors:** Rachel Yi Ling Kuo, Michael Ng, Daniel Prieto-Alhambra, Dominic Furniss

**Affiliations:** Oxford, United Kingdom; 1 From the Oxford National Institute for Health Research Musculoskeletal Biomedical Research Unit, Nuffield Department of Orthopaedics, Rheumatology, and Musculoskeletal Sciences, and the Department of Plastic and Reconstructive Surgery, Nuffield Orthopaedic Centre, University of Oxford; and the Oxford University Clinical Academic Graduate School, John Radcliffe Hospital.

## Abstract

Dupuytren’s disease is a common, chronic, fibroproliferative disease of the palmar fascia. The cause is unclear but includes genetic predisposition alongside environmental factors. Several studies have suggested an association between Dupuytren’s disease and excess mortality. The authors aimed to evaluate this association in adult patients in the United Kingdom and identify the causes of mortality.

**Methods:**

The authors used a large primary care database (Clinical Practice Research Datalink) linked to the Office of National Statistics to identify patients with Dupuytren’s disease between January 1, 1995, and December 31, 2013. Each patient was matched by age, sex, and general practitioner to five control patients without the disease. Cox regression models were used to study the association between Dupuytren’s disease and all-cause and cause-specific mortality, adjusting for confounders.

**Results:**

A total of 41,965 Dupuytren’s disease patients and 209,825 control patients were identified. The all-cause mortality rate was increased in both unadjusted (hazard ratio, 1.48; 99% CI, 1.29 to 1.70; *p <* 0.0001) and multivariable adjusted (hazard ratio, 1.43; 99% CI, 1.25 to 1.65; *p <* 0.0001) models in patients with Dupuytren’s disease, 12 years after diagnosis. Excess mortality was secondary to a wide range of causes, including cancer (hazard ratio, 1.66; 99% CI, 1.27 to 2.17; *p <* 0.0001), an effect that persisted after adjustment for confounders.

**Conclusions:**

There is excess mortality associated with Dupuytren’s disease that can be partially explained through environmental factors. From time of diagnosis in primary care, there is a 12-year window of opportunity for intervention to reduce the impact of these factors. The authors observed an increased risk of cancer mortality independent of confounders, and hypothesize a shared genetic risk between Dupuytren’s disease and cancer.

**CLINICAL QUESTION/LEVEL OF EVIDENCE:**

Risk, II.

## Abstract 2

Supplemental Digital Content is available in the text.

Dupuytren’s disease is a benign, progressive fibroproliferative disease of the palmar fascia that results in flexion contractures of the involved digits and significant functional impairment. It is also associated with debilitating fibromatoses of the feet (Ledderhose disease) and the penis (Peyronie’s disease).^1,2^ Dupuytren’s disease is very common, affecting 5 percent to 30 percent of people in populations of European descent.^3,4^ In the United Kingdom in 2004, the incidence of new consultations with general practitioners for Dupuytren’s disease was 34.3 per 100,000 men/year, and there is evidence that the prevalence is increasing.^5,6^

A genetic predisposition to Dupuytren’s disease is well recognized.^7–9^ There is also a clear nongenetic influence on development of the disease^10^: reported risk factors in hospital-based case-control studies include hypercholesterolemia,^11^ smoking and alcohol intake,^12^ diabetes,^13^ and epilepsy.^14^ Rheumatoid arthritis and increasing body mass index are thought to be protective against developing the disease.^15,16^

Patients with Dupuytren’s disease have also been reported to have a higher mortality rate than unaffected control subjects in small studies from Scandinavia and Iceland.^17–19^ This may be accounted for by the aforementioned nongenetic risk factors, but these studies were too small to answer this question. Despite being benign, Dupuytren’s disease shares many clinical and cell biological features with cancer, namely, increased cell proliferation, formation of tumor-like nodules, and the propensity to local recurrence after excision. Furthermore, a Swedish study has suggested a higher cancer incidence in patients with the disease.^20^ It is feasible that beyond the known common risk factors for Dupuytren’s disease and cancer, other factors (for example, genetic variants predisposing to the disease) might also predispose to certain types of cancer and again lead to an excess mortality rate in those affected.

The aim of this study was to identify any excess mortality associated with the diagnosis of Dupuytren’s disease compared with matched control patients in a large prospective cohort within the United Kingdom, and to quantify the contribution of environmental risk factors to mortality.

## METHODS

### Ethical Approval

This study was approved by the Independent Scientific Advisory Committee for Medicines and Healthcare Products Regulatory Agency Database Research (protocol 14_196R).

### Data Sources and Study Participants

We analyzed data from the United Kingdom Clinical Research Practice Datalink, a primary care database containing the anonymized health records of approximately 6.9 percent of the U.K. population,^21^ for the period between January 1, 1995, and December 31, 2013. Subjects were eligible for inclusion after completing 1 year of registration in a Clinical Research Practice Datalink–contributing, research-quality general practice. Dupuytren’s disease–exposed subjects were identified according to a Read code list defined independently by two clinically qualified researchers (R.K. and D.F.), with consensus reached by a third (D.P.-A.). (**See Table, Supplemental Digital Content 1**, which lists of all Read codes used to identify cases of Dupuytren’s disease within the Clinical Research Practice Datalink database and all risk factors studied, including smoking, diabetes mellitus, rheumatoid arthritis, epilepsy, hypercholesterolemia, hypertriglyceridemia, and alcohol consumption, http://links.lww.com/PRS/D952.) We excluded patients who did not have a recorded gender and those who were less than 18 or over 109 years of age on the date of diagnosis. The date of diagnosis was defined as the date that the patient had his or her first consultation with a general practitioner regarding Dupuytren’s disease.

We matched each Dupuytren’s disease participant to five disease-free control patients, who were defined as those never having been diagnosed with Dupuytren’s disease at the time of data extraction (August 25, 2015). Control patients were matched by age (±2 years), sex, and general practitioner’s practice.

Data extracted from the U.K. Clinical Research Practice Datalink included demographic details and diagnostic and therapeutic data regarding potential confounding factors, namely, body mass index, smoking and alcohol status, diabetes, epilepsy, rheumatoid arthritis, hypertriglyceridemia, and hypercholesterolemia. Previously validated code lists were identified for smoking status, alcohol use, diabetes mellitus, epilepsy, and rheumatoid arthritis. (**See Document, Supplemental Digital Content 2**, which contains a list of references that were used to identify previously validated code lists for risk factors, http://links.lww.com/PRS/D953.) A code list was generated for Dupuytren’s disease, hypercholesterolemia, and hypertriglyceridemia, as described above. Information regarding mortality rate was extracted using data linkage from the Office of National Statistics.

### Outcomes

Our primary outcome measure was all-cause mortality within the study period in cases of Dupuytren’s disease compared with matched controls. Our secondary outcome measures included 10 subsets of causes of death as defined using the *International Statistical Classification of Diseases and Related Health Problems, Tenth Revision*, namely, neoplastic, circulatory, respiratory, hematological, endocrine, psychiatric, genitourinary, musculoskeletal, digestive organ, and central and peripheral nervous system diseases. We then planned to analyze each significantly associated subgroup further to identify specific causes of excess mortality.

### Follow-Up

Our mortality model defined time-at-risk as the period between an index date until date of death, or end of follow-up (discharge from their general practitioner, or January 1, 2014), whichever came first. We defined an index date as being the date of diagnosis of Dupuytren’s disease for cases. The index date for control patients was defined as the date of diagnosis of Dupuytren’s disease for their matched case.

### Statistical Analyses

We used Stata MP 14.1 software (StataCorp, LLC, College Station, Texas) for all analyses. We reported all results using confidence intervals and *p* values at the 1% significance level. Within the U.K. Clinical Research Practice Datalink database, smoking status is stratified into current smoker, previous smoker, and never smoker. For each participant, we extracted the most recent recorded status prior to the index date. Alcohol exposure is similarly stratified and was extracted in an analogous way. Body mass index was stratified into three groups: less than or equal to 25 mg/m^2^, 25.1 to 29.9 kg/m^2^, and greater than or equal to 30 kg/m^2^.

We accounted for missing data by imputation using multivariate imputation by chained equations. We imputed data on smoking status, alcohol status, body mass index, and ethnicity. We created 10 imputed datasets using the following predictors for imputation: age at index date, sex, ethnicity, geographic region, Dupuytren’s disease status, smoking status, alcohol status, body mass index, diabetes, and death from cancer, cardiovascular disease, chronic lower respiratory tract disease, or liver disease. We combined the imputed datasets using Rubin’s rules. Due to a high proportion of missing data for alcohol consumption (81.1 percent of cases and 86.5 percent of controls, Table 1), we did not see convergence of datasets within multivariate imputation by chained equations (*p <* 0.0001, chi-square test). We did not, therefore, include alcohol in the final analyses, although we performed a sensitivity analysis with and without these missing data. (**See Table, Supplemental Digital Content 3**, which shows all-cause mortality rates in a multivariate-adjusted model, comparing hazard ratios including and excluding missing data for smoking and alcohol intake. This Table shows a sensitivity analysis for inclusion and exclusion of patients without smoking data and alcohol data, http://links.lww.com/PRS/D954.)

**Table 1.** Baseline Characteristics According to Dupuytren’s Disease Status in the U.K. Clinical Research Practice Datalink Database from 1995 to 2013

	No DD (*n* = 209,825)	DD (*n* = 41,965)	Odds Ratio (99% CI)	*p*
Mean age* (SD), yr	62.8 (12.0)	62.8 (12.0)	—	—
Body mass index			—	—
Mean value† (SD), kg/m^2^	27.4 (5.1)	26.7 (4.7)		
Missing (%)	118,900 (56.7)	10,898 (26.0)		
Alcohol consumption* (%)			—	—
Never	802 (0.4)	209 (0.5)		
Past	7129 (3.4)	899 (2.1)		
Current	20,373 (9.7)	6806 (16.2)		
Missing	181,521 (86.5)	34,051 (81.1)		
Smoking status* (%)			2.21 (2.14–2.27)‡	<0.0001
Never	50,965 (48.3)	16,734 (39.9)		
Past	29,089 (13.9)	10,420 (24.8)		
Current	21,365 (10.2)	6836 (16.3)		
Missing	108,406 (51.2)	7975 (19.0)		
Ethnicity (%)			—	—
Caucasian	35,112 (16.7)	7989 (19.0)		
Not Caucasian	2091 (1.0)	236 (0.6)		
Missing	172,622 (82.3)	33,740 (80.4)		
History of diabetes mellitus (%)	27,133 (12.9)	7074 (16.9)	1.37 (1.31–1.42)	<0.0001
History of rheumatoid arthritis (%)	3522 (1.7)	731 (1.7)	1.04 (0.93–1.15)	0.36
History of epilepsy (%)	3815 (1.8)	836 (2.00)	1.10 (0.99–1.21)	0.02
History of hypercholesterolemia or hypertriglyceridemia (%)	19,321 (9.2)	4801 (11.4)	1.27 (1.22–1.33)	<0.0001

DD, Dupuytren’s disease.

*At the date of diagnosis of Dupuytren’s disease for cases and the matched date for controls; odds ratio for alcohol consumption and ethnicity was not performed due to high percentage of missing data.

†Most recent body mass index measurement at time of, or most recent to, diagnosis of Dupuytren’s disease, calculated as weight in kilograms divided by height in meters squared.

‡Odds ratio reported for ever smokers/never smokers.

We used logistic regression to report odds ratios between cases of Dupuytren’s disease and their matched control cases for potential confounders defined from the literature, namely, body mass index, smoking, alcohol, diabetes, hypercholesterolemia and hypertriglyceridemia, epilepsy, and rheumatoid arthritis. When odds ratios were found to be significantly increased in Dupuytren’s disease compared with Dupuytren’s disease–free patients, they were included in the multivariable model.

We used Cox proportional hazards models stratified by matched sets to calculate hazard ratios according to Dupuytren’s disease status. We evaluated the effect of the disease on all-cause mortality and our predefined list of secondary outcomes. We tested the proportional hazards assumption using Schoenfeld’s residuals. Where this was violated, we analyzed survival in sections determined by the time point where there was deviation from proportionality, using the method of episode splitting to address time-varying covariates. This method is recommended as a clear and simple approach to nonproportionality in the event that only one covariate, or a few covariates, is nonproportional.^22^

We did not adjust for age, sex, or location, as these variables were matched for in our selection of control patients. We performed stepwise adjustment of our model using the following confounders: smoking status, diabetes mellitus, body mass index, hypercholesterolemia, and hypertriglyceridemia. Our final multivariable model included only smoking, body mass index, and diabetic status. We stratified patients with categorical diagnoses into three groups (diagnosed prior to the index date, diagnosed after the index date, or never diagnosed) and also evaluated the effect of body mass index and smoking exposure (defined above) on our primary and secondary outcomes.

## RESULTS

### Baseline Characteristics

We identified 41,965 eligible patients with Dupuytren’s disease and matched them to 209,825 control patients. There were 41,438 observed deaths during follow-up, 6016 of which occurred in Dupuytren’s disease patients.

Patients with Dupuytren’s disease were more likely to have a poor cardiovascular risk profile, including a history of current/past smoking (odds ratio, 2.21; 99% CI, 2.14 to 2.27; *p <* 0.0001), diabetes mellitus (odds ratio, 1.37; 99% CI, 1.31 to 1.42; *p <* 0.0001), and hypercholesterolemia or hypertriglyceridemia (odds ratio, 1.27; 99% CI, 1.22 to 1.33; *p <* 0.0001). They were no more likely to have a diagnosis of epilepsy than control patients. We found no protective association between rheumatoid arthritis and Dupuytren’s disease. Compared with participants with body mass index of 25 kg/m^2^ or less, other participants were less likely to have Dupuytren’s disease.

There was a high level of missing data for ethnicity (80.4 percent for Dupuytren’s disease patients and 82.3 percent for controls). This is similar to previously published literature on the completeness of ethnicity data within the U.K. Clinical Research Practice Datalink database.^23^ There have previously been observed differences in mortality rates among ethnic groups; however, this effect has been attributed to differences in socioeconomic status, body mass index, and health behaviors (e.g., smoking and alcohol).^24,25^ We accounted for these factors in our multivariable model.

### Mortality

**Fig. 1.**
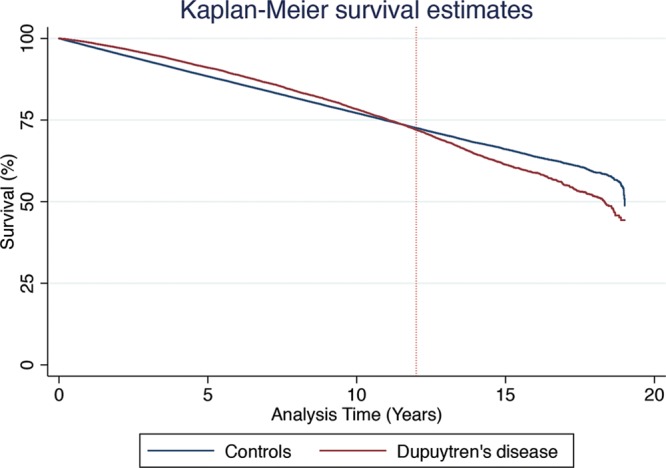
Kaplan-Meier survival curve of all-cause mortality comparing individuals with and without a diagnosis of Dupuytren’s disease from the date of diagnosis.

Table 2 summarizes the association between mortality rate and diagnosis of Dupuytren’s disease. We found that all-cause mortality hazard was nonproportional, changing at 12 years after diagnosis (Fig. 1). The presence or absence of Dupuytren’s disease was found to be the only covariate that violated the proportional hazards assumption. This pattern was observed in most subsets of mortality. We therefore split our analysis into two sections: the first for mortality within 12 years of disease diagnosis, and the second for mortality thereafter. There were three exceptions to this observation: death secondary to liver disease and death caused by lip, oral, and pharyngeal cancer did not violate the proportional hazards assumption; death caused by suicide or self-harm violated the proportional hazards assumption at 5 years (Table 3).

**Table 2.** Unadjusted and Multivariable Adjusted Hazard Ratios (99% Confidence Intervals) for Mortality According to Dupuytren’s Disease Status in the U.K. Clinical Research Practice Datalink Database, 1995 to 2013: Main ICD-10 Categories*

Cause of Death (no. of deaths)	0 to 12 Years		12 to 20 Years	
	DD HR (99% CI)	*p*	DD HR (99% CI)	*p*
All-cause mortality (*n* = 41,438)				
Unadjusted	0.82 (0.79–0.85)	<0.0001	1.48 (1.29–1.70)	<0.0001
Multivariable adjusted**	0.80 (0.77–0.83)	<0.0001	1.43 (1.25–1.65)	<0.0001
Cancer (*n* = 8098)				
Unadjusted	0.90 (0.83–0.98)	0.0013	1.66 (1.27–2.17)	<0.0001
Multivariable adjusted	0.86 (0.79–0.94)	<0.0001	1.63 (1.24–2.14)	<0.0001
Cardiovascular disease (*n* = 8225)				
Unadjusted	0.86 (0.79–0.94)	<0.0001	1.47 (1.13–1.91)	0.0002
Multivariable adjusted	0.80 (0.73–0.87)	<0.0001	1.38 (1.05–1.80)	0.0021
Respiratory disease (*n* = 3427)				
Unadjusted	0.88 (0.77–1.00)	0.0103	1.64 (1.14–2.38)	0.0005
Multivariable adjusted	0.80 (0.70–0.92)	<0.0001	1.50 (1.03–2.19)	0.0051
Digestive organ disease (*n* = 1177)				
Unadjusted	1.08 (0.88–1.33)	0.3438	2.32 (1.22–4.41)	0.0007
Multivariable adjusted	1.00 (0.81–1.23)	0.9924	2.12 (1.11–4.05)	0.0028
Endocrine disease (*n* = 343)				
Unadjusted	1.18 (0.80–1.70)	0.3027	3.32 (1.21–9.08)	0.0021
Multivariable adjusted	0.80 (0.54–1.17)	0.1285	2.34 (0.84–6.54)	0.0327
Psychiatric disease (*n* = 1072)				
Unadjusted	0.98 (0.77–1.23)	0.7864	1.81 (1.06–3.09)	0.0040
Multivariable adjusted	0.90 (0.71–1.14)	0.2605	1.70 (0.99–2.92)	0.0113

ICD-10, *International Statistical Classification of Diseases and Related Health Problems, Tenth Revision*; DD, Dupuytren’s disease; HR, hazard ratio.

*Multivariable models were adjusted for diabetes mellitus (diagnosis before Dupuytren’s disease/after Dupuytren’s disease/never), smoking status (current/ex-smoker/never smoker, most recent status prior to Dupuytren’s disease diagnosis), and body mass index (≤25 kg/m^2^, 25 to 30 kg/m^2^, >30 kg/m^2^).

**Table 3.** Unadjusted and Multivariable Adjusted Hazard Ratios for Mortality According to Dupuytren’s Disease Status in the U.K. Clinical Research Practice Datalink Database, 1995 to 2013: Subcategories of ICD-10 Classification*

Subcategory (no. of deaths)	0 to 12 Years (*n* =251,790)		12 to 20 Years (*n* =31,990)	
	DD (*n* =41,965) HR (99% CI)	*p*	DD (*n* =4544) HR (99% CI)	*p*
Lung cancer (*n* = 1762)				
Unadjusted	0.95 (0.79–1.13)	0.4103	1.76 (0.95–3.28)	0.0184
Multivariable adjusted	0.91 (0.76–1.08)	0.1606	1.77 (0.95–3.31)	0.0191
Prostate cancer (*n* = 766)				
Unadjusted	0.94 (0.71–1.23)	0.5339	1.86 (0.87–3.95)	0.0347
Multivariable adjusted	0.88 (0.67–1.17)	0.2671	1.79 (0.84–3.83)	0.0484
Lip, oral, and pharyngeal cancer (*n* = 100)†				
Unadjusted	2.10 (1.18–3.73)	0.0009	2.10 (1.18–3.73)	0.0009
Multivariable adjusted	1.41 (0.64–3.16)	0.2599	1.41 (0.64–3.16)	0.2599
Digestive organs cancer (*n* = 2378)				
Unadjusted	0.91 (0.78–1.06)	0.1179	1.66 (1.01–2.72)	0.0087
Multivariable adjusted	0.87 (0.74–1.01)	0.0189	1.60 (0.97–2.63)	0.0160
Cerebrovascular disease (*n* = 2012)				
Unadjusted	0.85 (0.72–1.12)	0.0189	1.72 (1.03–2.87)	0.0065
Multivariable adjusted	0.79 (0.67–0.94)	0.0006	1.57 (0.94–2.65)	0.0230
Ischemic heart disease (*n* = 4138)				
Unadjusted	0.88 (0.78–0.99)	0.0048	1.54 (1.05–2.27)	0.0035
Multivariable adjusted	0.80 (0.71–0.90)	<0.0001	1.40 (0.95–2.06)	0.0267
Chronic lower airway disease (*n* = 1439)				
Unadjusted	0.80 (0.65–0.98)	0.0056	1.79 (0.88–3.62)	0.0347
Multivariable adjusted	0.76 (0.61–0.96)	0.0023	1.68 (0.72–3.88)	0.1123
Liver disease (*n* = 326)†				
Unadjusted	1.83 (1.32–2.54)	<0.0001	1.83 (1.32–2.54)	<0.0001
Multivariable adjusted	2.03 (1.32–3.12)	<0.0001	2.03 (1.32–3.12)	<0.0001
Diabetes (*n* = 273)				
Unadjusted	1.18 (0.77–1.81)	0.3121	3.14 (1.03–9.59)	0.0082
Multivariable adjusted	0.75 (0.49–1.15)	0.0808	2.08 (0.67–6.47)	0.0975
Suicide and self-harm (*n* = 105)‡				
Unadjusted	0.83 (0.33–2.08)	0.5958	3.76 (1.64–8.63)	<0.0001
Multivariable adjusted	0.80 (0.32–2.03)	0.5436	3.73 (1.61–8.64)	0.0001

ICD-10, *International Statistical Classification of Diseases and Related Health Problems, Tenth Revision*; DD, Dupuytren’s disease; HR, hazard ratio.

*Multivariable models were adjusted for diabetes mellitus (diagnosis before Dupuytren’s disease/after Dupuytren’s disease/never), smoking status (current/ex-smoker/never smoker, most recent status prior to Dupuytren’s disease diagnosis), and body mass index (≤25 kg/m^2^, 25 to 30kg/m^2^, >30 kg/m^2^).

†Proportional hazards assumption was not violated.

‡Violated proportional hazards assumption at 5 years after diagnosis.

We observed a delayed increase in mortality rate for Dupuytren’s disease patients compared with Dupuytren’s disease–free subjects. There was a small survival benefit (hazard ratio, 0.82; 99% CI, 0.79 to 0.85; *p <* 0.0001) in the first 12 years after diagnosis of Dupuytren’s disease, followed by increased mortality (hazard ratio, 1.48; 99% CI, 1.25 to 1.65; *p <* 0.0001) beyond 12 years.

Specifically, after 12 years, we found increased mortality secondary to cancer (hazard ratio, 1.6; 99% CI, 1.27 to 2.17; *p <* 0.0001), cardiovascular disease (hazard ratio, 1.47; 99% CI, 1.13 to 1.91; *p* =0.0002), respiratory disease (hazard ratio, 1.64; 99% CI, 1.14 to 2.38; *p* = 0.0005), diseases of the digestive organs (hazard ratio, 2.32; 99% CI, 1.22 to 4.41; *p* =0.0007), endocrine disease (hazard ratio, 3.32; 99% CI, 1.21 to 9.08; *p* =0.0021), and psychiatric disease, including suicide and self-harm (hazard ratio, 1.81; 99% CI, 1.06 to 3.09; *p* =0.0040).

Subgroup analysis within these categories (Table 3) revealed that patients with Dupuytren’s disease were more likely to die from lung, digestive organ, and lip, oral, and pharyngeal cancer. Furthermore, we found that increased mortality secondary to respiratory disease was likely to be caused by chronic lower airway disease. Within the group of patients who died from cardiovascular disease, the major contributors to excess death were cerebrovascular disease and ischemic heart disease. Death secondary to diabetes was responsible for the excess death from endocrine causes. Patients with Dupuytren’s disease were also more likely to die from liver disease throughout the course of follow-up. Finally, they were more likely to die from suicide or self-harm, with this increased risk starting at 5 years from the case index date.

We explored preliminary evidence for the reasons behind the increased mortality within our dataset by stepwise multivariable regression to account for known common risk factors for both Dupuytren’s disease and mortality: smoking status, body mass index, and diabetes mellitus. In some cases, adjustment for diabetes, smoking status, and body mass index within our regression model eliminated the excess mortality (Tables 2 and 3). However, even using a multivariable model adjusting for these confounders, patients with Dupuytren’s disease had a significant increase in all-cause mortality (hazard ratio, 1.43; 99% CI, 1.25 to 1.65; *p <* 0.0001), cancer-related mortality (hazard ratio, 1.63; 99% CI, 1.24 to 2.14; *p <* 0.0001), mortality caused by cardiovascular disease (hazard ratio, 1.38; 99% CI, 1.05 to 1.80; *p* =0.0021), and mortality caused by respiratory disease (hazard ratio, 1.50; 99% CI, 1.03 to 2.19; *p* =0.0051) beginning 12 years after diagnosis. Adjusting for diabetes and smoking status did not eliminate the risk of death from liver disease throughout follow-up (hazard ratio, 2.03; 99% CI, 1.32 to 3.12; *p <* 0.0001) or from suicide and self-harm (hazard ratio, 3.73; 99% CI, 1.61 to 8.64; *p* =0.0014) starting from 5 years after diagnosis.

### Missing Data Sensitivity Analysis

There was a moderate level of missing data for smoking (46.2 percent) and a high level of missing data for alcohol consumption (85.6 percent) (Table 1). We performed a sensitivity analysis first including cases and controls with missing data and second after removal of cases and controls with missing data for both smoking and alcohol consumption. In all analyses, the results remained consistent, demonstrating reduced mortality from diagnosis until 12 years and increased mortality after 12 years. (**See Table, Supplemental Digital Content 3**, which shows all-cause mortality in a multivariate-adjusted model, comparing hazard ratios including and excluding missing data for smoking and alcohol intake. This Table shows a sensitivity analysis for inclusion and exclusion of patients without smoking data and alcohol data, http://links.lww.com/PRS/D954.)

## DISCUSSION

Our study has demonstrated an increased risk of death in patients with Dupuytren’s disease, a very common fibroproliferative disorder of the hand. This excess mortality is accounted for by increase in death from cancer, cardiovascular disease, diabetes, liver disease, and suicide. Importantly, for most causes of excess mortality, there is a delay of 12 years before this increase becomes evident, representing an important therapeutic window for intervention by healthcare professionals. Adjusting for smoking and diabetic status in our multivariable regression model reduced the effect size of increased mortality in patients with Dupuytren’s disease, which suggests that these factors are at least partially responsible. We recommend that special attention be paid to smoking cessation, control of diabetes, and dyslipidemia management in this group of patients when they first present to medical services. We hypothesize that there is a 12-year delay in increased mortality secondary to the nature of the causes of death that we have identified. For example, it may take 12 years for a poor cardiovascular profile to manifest into a life-threatening myocardial infarction.

Intriguingly, we found that there remained an increase in all-cause and cancer-related mortality after adjustment for confounding factors associated with both Dupuytren’s disease and mortality. Dupuytren’s disease is a complex genetic disease, with multiple genetic loci interacting with environmental factors, leading to the disease phenotype. A previous genome-wide association study identified nine genetic loci associated with Dupuytren’s disease, of which six were involved in the WNT signaling pathway,^26^ a family of proteins that regulate cell growth, differentiation, and proliferation. Deregulation of WNT signaling is implicated in the development of multiple cancers, including colorectal, breast, prostate, and oropharyngeal cancer.^27–29^ It is conceivable that there may be a shared genetic predisposition to both cancer and Dupuytren’s disease, and this hypothesis deserves further study.

We confirmed previous studies that have demonstrated an association between Dupuytren’s disease and smoking.^17^ The effects of this association are reflected in the increased risk of death from respiratory disease, alongside lip, oral, and pharyngeal cancer. We also found that patients with Dupuytren’s disease had an unfavorable cardiovascular profile compared with matched controls: in addition to smoking, they were more likely to have a diagnosis of diabetes and hypercholesterolemia and hypertriglyceridemia. As with respiratory disease, we found that adjusting for smoking status accounted for some of the observed increased cardiovascular mortality. Interestingly, adjusting for a prior diagnosis of diabetes or an elevated lipid profile in our multivariable model reduced, but did not eliminate, the observed increased risk of death from cardiovascular disease or its subsets.

We could not include alcohol consumption in our multivariable model due to a large proportion of missing data and an inability to accurately impute these data. However, liver disease was a consistent cause of increased mortality and suggests some shared etiology. Intriguingly, we have also discovered a novel finding of increased mental illness among patients with Dupuytren’s disease. In particular, patients were more likely to die from suicide than their matched controls, beginning 5 years after diagnosis. We suggest that alcohol-associated Dupuytren’s disease may account for increased mortality secondary to liver disease, and may also be a causative factor in patients’ poorer mental health.

Our study has several strengths, including its large sample size, spanning primary care practices across the United Kingdom. Basing the study in primary care increases the generalizability of our findings compared with previous studies that have focused on Dupuytren’s disease in secondary care, for example, by including only those who have had surgery for Dupuytren’s disease. This is the largest study to date assessing the mortality associated with Dupuytren’s disease, encompassing both genders, all ethnicities, and all age groups. Our study populations had similar baseline characteristics: matching was performed on age, sex, and location. A further strength of our study lies in its comprehensive assessment of comorbidities that have been associated with Dupuytren’s disease in the literature. Our large sample size provided adequate power to assess their individual and combined effects on mortality.

However, several limitations must be taken into account. As with any observational study, unmeasured confounding could be responsible for the effects seen. Our work relies on clinical coding of Dupuytren’s disease and comorbidities in general practice, and inaccurate coding could lead to misclassification bias. Coding of Dupuytren’s disease may not be at the actual onset of disease, and there is likely to be a lag between its development and eventual diagnosis in primary care. To address these sources of bias, we used matched controls and set their entry into the study at the time of diagnosis of Dupuytren’s disease. We could not account for severity of disease, which could introduce bias: patients are more likely to be coded as having Dupuytren’s disease if it is more severe. We were unable to incorporate alcohol use into our multivariable model due to a high proportion of missing data and inability to impute this data. Alcohol use has traditionally been associated with development of Dupuytren’s disease and is associated with increased mortality. Finally, we did not assess the impact of medications on mortality, including control of diabetes mellitus and the use of statins in lipid regulation.

## CONCLUSIONS

Physicians, general practitioners, and surgeons treating patients with Dupuytren’s disease should recognize its presence as a sign of poor general health and act to modify risk factors for mortality in their patients. There is an unfavorable outlook with regard to cancer, cardiovascular disease, respiratory disease, endocrine disturbance, liver pathology, and mental health. We have shown that several of the contributing causes of death in this cohort are in part attributable to smoking status, these being cancer, chronic lower respiratory tract disease, and cardiovascular disease. Importantly, this study has identified a 12-year window of opportunity for clinical and lifestyle intervention to avoid premature mortality, particularly with regard to smoking cessation and treatment of diabetes and hyperlipidemia.

## ACKNOWLEDGMENTS

Professor Furniss was supported by an Intermediate Clinical Fellowship from the Wellcome Trust (097152/Z/11/Z). This work and its research were supported by the National Institute for Health Research Oxford Biomedical Research Centre. Dr. Prieto-Alhambra is funded through a National Institute for Health Research Senior Research Fellowship (grant SRF-2018-11-ST2-004). The authors acknowledge Alice Fuller and Sarah Stevens, Nuffield Department of Primary Health Care Sciences, University of Oxford, for help with data management. They thank Gary Collins, Nuffield Department of Orthopaedics, Rheumatology, and Musculoskeletal Science, University of Oxford, for critical review of the manuscript.

## DISCLAIMER

The views expressed in this publication are those of the authors and not necessarily those of the National Health Service, the National Institute for Health Research, or the Department of Health.

## Supplementary Material

SUPPLEMENTARY MATERIAL
