# Supplementary material for: Dupuytren’s Disease Predicts Increased All-Cause and Cancer-Specific Mortality: Analysis of a Large Cohort from the U.K. Clinical Practice Research Datalink
Source: Plast Reconstr Surg. 2019 Dec 17;145(3):574–82. doi: 10.1097/PRS.0000000000006551 (PMC7043723; doi:10.1097/PRS.0000000000006551)
Supplement: SUPPLEMENTARY MATERIAL [file prs-145-574e-s001.pdf]

Dupuytren’s disease codes	
**readcode**	**readterm**
**7H3A000**	Dermofasciectomy
**7H32800**	Digital fasciectomy
**7H34011**	Division of hand fascia
**7H34000**	Division of palmar fascia
**7H32011**	Dupuytren hand fasciectomy
**7H34012**	Dupuytren hand fasciotomy
**N236.00**	Dupuytren's contracture
**N236700**	"Dupuytren's dis, palm and finger(s), nodules, no contracture"
**N236400**	"Dupuytren's disease - finger(s), nodules with no contracture"
**N236300**	Dupuytren's disease of finger(s)
**N236500**	"Dupuytren's disease of finger(s), with contracture"
**N236000**	Dupuytren's disease of palm
**N236600**	Dupuytren's disease of palm and finger(s)
**N236800**	"Dupuytren's disease of palm and finger(s), with contracture"
**N236100**	"Dupuytren's disease of palm, nodules with no contracture"
**N236200**	"Dupuytren's disease of palm, with contracture"
**7H35700**	Fasciotomy hand
**7H34z11**	Fasciotomy NEC
**7H32012**	Hand fasciectomy
**7H32400**	Limited palmar fasciectomy
**7H32013**	McIndoe radical palmar fasciectomy
**7H34300**	Needle fasciotomy of hand
**N236.11**	Palmar fascia contracture
**7H32000**	Palmar fasciectomy unspecified
**7H32700**	Palmar fasciectomy using open palm technique
**7H32500**	Radical palmar fasciectomy
**7H32900**	Revision of digital fasciectomy
**7H32100**	Revision of palmar fasciectomy
