# Supplementary material for: Dupuytren’s Disease Predicts Increased All-Cause and Cancer-Specific Mortality: Analysis of a Large Cohort from the U.K. Clinical Practice Research Datalink
Source: Plast Reconstr Surg. 2019 Dec 17;145(3):574–82. doi: 10.1097/PRS.0000000000006551 (PMC7043723; doi:10.1097/PRS.0000000000006551)
Supplement: SUPPLEMENTARY MATERIAL [file prs-145-574e-s002.pdf]

Supplementary References

1. NA. Quality Outcomes Framework Business Rules v32.0

2. Kontopantelis E, Springate DA, Reeves D et al. Glucose, blood pressure and cholesterol levels and their relationships to clinical outcomes in type 2 diabetes: a retrospective cohort study. Diabetologia, dos:10.1007/s00125-014-3473-8

3. Carr MJ, Ashcroft DM, Kontopantelis E et al. Premature death among primary care patients with a history of self-harm. Ann Fam Med. 2017 May;15(3):246-254.doi: 10.1370/afm.2054

4. Nicholson A, Ford E, Davies KA et al. Optimising use of electronic health records to describe the presentation of rheumatoid arthritis in primary care: a strategy for developing code lists. PLoS ONE 2013, doi:10.1371/journal.pone.0054878
