# Supplementary material for: Dupuytren’s Disease Predicts Increased All-Cause and Cancer-Specific Mortality: Analysis of a Large Cohort from the U.K. Clinical Practice Research Datalink
Source: Plast Reconstr Surg. 2019 Dec 17;145(3):574–82. doi: 10.1097/PRS.0000000000006551 (PMC7043723; doi:10.1097/PRS.0000000000006551)
Supplement: SUPPLEMENTARY MATERIAL [file prs-145-574e-s003.pdf]

		Hazard Ratio (99% CI)					
		0 - 12 years			12 - 20 years		
**Outcome (mortality)**		**No Dupuytren's disease**	**Dupuytren's disease**		**No Dupuytren's disease**	**Dupuytren's disease**	**p value**
All-cause mortality	Including missing smoking and alcohol data n = 251790	REF	0.83 (0.80 - 0.87)	<0.0001	REF	1.54 (1.37 - 1.73)	<0.0001
	Excluding missing smoking data n = 135409	REF	0.81 (0.78 - 0.85)	<0.0001	REF	1.45 (1.15 - 1.82)	<0.0001
	Excluding missing alcohol data n= 36218	REF	0.80 (0.73 - 0.88)	<0.0001	REF	1.83 (1.21 - 2.79)	0.0024
Multivariate models are adjusted for diabetes mellitus (diagnosis prior to Dupuytren's disease/after Dupuytren's disease/never), and smoking status (current/ex-smoker/never smoker, most recent status prior to Dupuytren's disease diagnosis)							
